# Us helping us: The evolution of a peer support group for formerly incarcerated people

**DOI:** 10.3389/fpsyt.2022.920640

**Published:** 2022-08-02

**Authors:** Will Boles, Thad Tatum, Jarrod Wall, Lauren Nguyen, Alexandria Van Dall, Claire Mulhollem, Anna Sacks, Ashley Wennerstrom, Bruce Reilly, Anjali Niyogi

**Affiliations:** ^1^Louisiana State University Health Sciences Center New Orleans School of Medicine, and Harvard Kennedy School of Government, New Orleans, LA, United States; ^2^Formerly Incarcerated Peer Support (FIPS) Group, New Orleans, LA, United States; ^3^City, Culture, and Community Program, Tulane University, New Orleans, LA, United States; ^4^Tulane University School of Public Health and Tropical Medicine, New Orleans, LA, United States; ^5^Formerly Incarcerated Peer Support (FIPS) Group, New Orleans, LA, United States; ^6^Department of Sociology, School of Liberal Arts, Tulane University, New Orleans, LA, United States; ^7^Voice of the Experienced (VOTE), New Orleans, LA, United States; ^8^Behavioral and Community Health Sciences, School of Public Health, New Orleans, LA, United States; ^9^Internal Medicine and Pediatrics, Tulane University School of Medicine, New Orleans, LA, United States

**Keywords:** incarceration, reentry, peer support, mental health, program development, lived experience, community

## Abstract

**Introduction:**

Physical, psychological, and emotional trauma experienced while incarcerated influences subsequent mental health outcomes. Upon release, there is a fragmented landscape of mental health services and many of the existing services do not account for the root causes of challenges faced by formerly incarcerated people (FIP). To address the unmet social, psychological, behavioral, and emotional needs of FIP in Louisiana, the Formerly Incarcerated Peer Support (FIPS) Group developed a twelve-unit curriculum in 2019.

**Methods:**

We detail the evolution, development, and evaluation of the FIPS Group program. Additionally, we describe the community-driven process for developing the curriculum.

**Results:**

The FIPS Group has grown from informal meetings of a handful of FIP in New Orleans, Louisiana, into a multi-state, interdisciplinary network of more than 150 stakeholders. FIPS Group has developed the only peer support curriculum we are aware of that is designed by FIP, for FIP, and uses the shared experience of incarceration and reentry as its organizing principle. Limitations of the model include the lack of pending evaluation data and challenges with technological proficiency among FIP.

**Conclusions:**

The FIPS Group model may be generalized in a number of settings. Similar approaches may benefit the mental health of the millions of Americans involved in the criminal-legal system.

## Introduction

The Formerly Incarcerated Transitions (FIT) Clinic was established in New Orleans, Louisiana in 2014 through a partnership between a local medical school and a grassroots organization of formerly incarcerated criminal justice reform advocates to meet the unique medical needs of formerly incarcerated people (FIP). While the clinic was successfully providing medical care and case management following release from incarceration, providers and case managers at the clinic recognized that mental health remained a significant area in need of growth (1).

One of the clinic’s first patients, Mr. J, experienced homelessness for more than 10 years. He had no family in town, was unemployed, and began coming to the clinic every day. Mr. J was first incarcerated at the age of 16. During his entire adult life, he only knew the confines and routines of a total institution. He struggled with hopelessness and suicidality. This emotional and behavioral distress was rooted in the lived experience of having first to adjust to incarceration, and then having to adjust to its absence. Facing the difficulty of navigating employment, housing, technology, and social stigmatization while trying to reconnect with family and former friends, Mr. J returned to a city he used to call home but no longer recognized. The FIT Clinic could care for his physical health but addressing this patient’s mental distress required someone who understood the institutions that had shaped so much of his life. It became clear to the clinic staff that to properly meet the immense need for care, a firsthand knowledge of United States prisons was just as necessary to treat FIT Clinic patients as any professional training.

The United States incarcerates more people than any other country in the world. There are roughly 70 million Americans who have been arrested, with varying lengths of incarceration (2). Most of the 2.3 million people actively incarcerated in the United States at any given time will be released, contributing to the approximately 4.4 million individuals on probation and parole (3, 4). Thus, most of those involved in the criminal-legal system in the United States are FIP. Louisiana has one of the highest rates of justice-involvement in the country, with an incarceration rate of 680 per 100,000, compared to a national average of 419 per 100,000 as of 2019 (5).

Incarceration is associated with the development of disability, both physical and cognitive, that adds social and economic stress to reintegration (6). FIP must deal with enduring stressors such as social stigma and disruption of bonds like employment and relationships with family and friends (7). Recently released individuals face greater health risks, with a 12-fold increase in mortality in the first 2 weeks following release (1, 8). This often takes the form of drug overdose, lack of continued care for chronic conditions like cardiovascular disease, and by violent victimization (9).

Applying a trauma-informed lens to experiences of incarceration provides important insight for understanding causal relationships between incarceration and mental and behavioral health (10, 11). Rates of potentially traumatic events (PTEs) during incarceration have been reported as high as 96.8% among incarcerated people (12). One study found that 89% of individuals witnessed or experienced an assault while incarcerated (13). Previous meta-analyses and systematic reviews have indicated that trauma from imprisonment has a stronger association with later mental disorders relative to other forms of trauma, such as in childhood or through one’s total lifetime, despite the fact that these points in the life-course are more well-studied (14). Additionally, though traumatic experiences in prison are positively associated with later PTSD outcomes, time spent incarcerated is not. This suggests that incarceration for any length of time places individuals at an increased risk for traumatic experiences, and thus negative mental health outcomes (15).

Beyond trauma, the mental distress of patients like Mr. J points toward the lasting effects of institutionalization. Institutionalization in prison may be defined as “the embodiment of inequities of incarceration” (16). Institutions like prisons subsume all aspects of personhood and agency, including decisions about self-care and wellbeing, relegating them to the goals of the institution housing them (17). As Crane and Pascoe poignantly elaborate: “The dehumanizing power structure within prisons and the broader inequities that shape mass incarceration manifest as the physical, mental, and social ailments that come with long-term imprisonment” (16). More concretely, incarcerated people develop necessary adaptive behaviors and cognitions, which are not pathological for their environment in prison, yet these behaviors can become internalized and maladaptive upon release. For example, counting the entrances to a room and keeping one’s back to the wall can enable one to negotiate the spontaneous violence that erupts in prisons, yet such behavior might be deemed hypervigilance and possibly paranoia upon release.

Aside from positive stressors of institutional environments, institutionalization involves adapting to the deprivation of meaningful activities. Incarcerated people do not decide what they wear, when or what they eat, or what they do with their time. In the United States, where both budget cuts and punitive policies limit access to enriching vocational, educational, and rehabilitative programming, many individuals experience “dead time.” Dead time can be understood as occupational deprivation, where incarcerated people are stripped not merely of agency in daily tasks, but of meaningful roles, such as being a mother or mechanic or son (18, 19).

Typical reentry services that acknowledge institutional behaviors as potential barriers to assimilation fail to recognize society’s complicity in recidivism. Prison programs designed to promote “desistance” of criminal activity are unable to account for the predisposing factors to success after release (20). Namely, that returning to racialized, exploited, low-income communities predisposes one to both initial imprisonment and subsequent recidivism (21). The experience of incarceration and its associated deprivations during and after release is a bigger handicap than any array of evidence-based, individual interventions can alleviate without decarcerating our society.

A significant factor impacting incarceration, both initially, and especially after reentry, is social exclusion (22, 23). Discrimination is legalized through policies in employment, housing, education, and voting, as Equal Protection (guaranteed to people without felonies, under the United States Constitution) no longer applies to many people convicted of felonies (24). A separate civil society is reinforced through the norms of a dominant culture which castigates and ostracizes FIP. Failures to navigate an apartheid system are then deemed as personal failings by the FIP, with hardly any recognition of barriers explicitly designed to prevent assimilation. These barriers range from barring people with felony convictions from dating apps to banning volunteering at the schools of a formerly incarcerated person’s own child (25–27). While some FIP-led organizations have been at the forefront of easing these barriers and promoting a single civil society, the hypocrisy of blaming FIP who fail to assimilate into a society constructed to prevent assimilation, may generate deep feelings of resentment, bitterness, hopelessness, and/or self-loathing.

The combined factors of institutionalization, post-release stigma, and discrimination leave lasting impacts on FIP after release. They may manifest as social isolation, hampered decision-making capacity, an inability to keep up with bills, social awkwardness, maladaptive communication skills, and other functional deficits (28, 29). Clinically, individuals may experience anxiety, depression and suicidality – just as Mr. J demonstrated (9).

Peers (i.e., other FIP) can have a significant positive impact on the reentry process for someone coming home. Through expressive, emotional, informational, and materially based means, peers are powerful in that they can provide a unique form of support to FIP that has been shown to reduce behaviors leading to recidivism (30, 31). Programs that have utilized peer reentry specialists have demonstrated that FIP leverage their lived experience to help others seek treatment for substance use and mental health needs, locate housing, and secure employment (32). This protective effect on recidivism is mediated through the strong bonds formed as a result of mutual trust between FIP and their peers (33, 34). However, meta-analyses have described wide variations in goals and execution of these interventions leveraging peer support models, and high-quality evidence is still forthcoming (15, 35, 36).

While studies support the need for trauma-informed care and mental health counseling during the reentry period, (37) there is little known about how a peer support program, particularly one led by FIP, could be developed and established. Many existing reentry services do not directly focus on mental and behavioral health. Meanwhile, many state-funded clinical services focus only on people with specific diagnoses, such as substance use disorders, rather than those with a history of justice-involvement. Thus, psychiatric, or clinical care is siloed from peer support services. To fill this gap and meet the needs of individuals like Mr. J, a collaboration between FIP and local stakeholders was undertaken to create a peer support group led by and for FIP. The present paper describes the development and implementation of a peer support group for FIP and offers suggestions for outcome and process evaluation.

## Context and development

### Establishing partnerships

Even though Louisiana has one of the highest rates of justice involvement in the United States, there was previously no mechanism or specialized resource for basic medical care for FIP in Louisiana after release. FIP were given no more support than a $10 check and a bus token upon release. An internal medicine physician (AN) established the FIT Clinic in 2014 to provide medical care to those transitioning out of incarceration through a partnership between a local medical school and a grassroots organization of formerly incarcerated criminal justice reform advocates (38, 39).

While providers at the FIT Clinic were seeing patients like Mr. J, a FIP trained in addiction counseling (TT) was working at a behavioral health center providing peer support services for people seeking substance use treatment and expanded the center’s work by recruiting justice-involved people. Having organized and led substance use peer support groups for 15 years at the Louisiana State Penitentiary (Angola), TT knew how to create spaces that were familiar, safe and therapeutic. Using those same skills, TT created a space that FIP would be familiar with and learned adapted the discussions from substance use to reentry and re-adjustment. As a result, the Formerly Incarcerated Peer Support (FIPS) Group was formed. Recruitment came by word-of-mouth from those who were also incarcerated at Angola.

### Early development and evaluation

As the FIPS Group grew, TT began working directly with a medical student who assisted the group’s development. Meetings began on the second and fourth Tuesday of each month, and from 2015 to 2017 they remained largely informal, peer-led sessions where participants could voice and process with others both their carceral and reentry experiences – something they were unable to find anywhere else. An early evaluation of the group was conducted in 2017, during its second year of operation (40). This study was deemed exempt by the Tulane University institutional review board. In September 2017, group participants were invited to complete the Client Satisfaction Questionnaire (CSQ-8). The CSQ-8 is a brief, unidimensional 8-item scale originally developed to measure client satisfaction in mental health programs. It has demonstrated very good to excellent internal consistency, based on tested values for coefficient alpha that range from 0.83 to 0.94 (41). Its correlation with variables such as program completion further supports its concurrent validity. Sixteen participants completed the surveys. Participants were 87.5% male, 100.0% Black, and 56.3% were aged 55 or older. At the time of evaluation, the average length of incarceration was 21.6 years and the average time since release was 6.6 years. Two-thirds of participants were referred to the group via word of mouth. CSQ-8 scores range from 8 to 32. Median CSQ-8 scores among survey participants was 30.5, indicating high satisfaction. Individual responses from the CSQ-8 found that the group rated high for likelihood of recommendation to a friend, intention to come back, and overall likelihood to come back, yet it rated relatively lower for feeling that their needs were met and that the group helped them deal with their problems.

### Curriculum development

With insight from the early evaluation, the FIPS Group began to formalize its organizational goals, and hired a consultant for training in reentry peer support. Grant-funded peer support specialist training from PeerStar, LLC was provided to a few FIPS Group members in 2019. PeerStar provides forensic peer support services to jails in Pennsylvania (42). This training provided more specific guidance on organizing and facilitating peer-led programs and encouraged formal, “classroom”-style methods for group implementation. However, based on informal focus group discussions, FIP felt that while the reentry curriculum provided life skills such as seeking employment and housing, it did not adequately address their emotional and psychosocial needs. The orientation of most reentry peer support or group-based mental health interventions is from that of carceral institutions. This traditional format may facilitate instrumental support, such as coordination with mental health services, but such interventions are ill-equipped to provide a safe space to unpack experiences of incarceration (43, 44). Experiences of incarceration and institutionalization were identified by FIPS Group organizers as the root cause of much of their and other FIP’s physical, psychological, and behavioral challenges. This led to the development of a novel curriculum, organized around the shared experiences of incarceration, similar to Crane and Pascoe’s conception of incarceration as a chronic condition (15).

The specific needs of FIP led to the development of a novel facilitation strategy coinciding with curriculum development. Recognizing that organic conversation functioned as the primary engine of the community-building and solidarity among FIPS Group members, organizers decided to move forward with their existing method of semi-structured and relaxed facilitation. This method also abstained from psychoeducational techniques due to the value placed on flexible, non-hierarchical group dynamics. For example, rather than formally instructing coping skills, the objective was to enable group members to implicitly learn how to adapt to post-carceral life from one another. Thus, FIPS Group operates primarily as a form of an interpersonal process group, providing interpersonal learning, mutual support, developing post-carceral socializing techniques, and establishing a safe space, analogous to those services offered to veterans (44–46).

Formerly Incarcerated Peer Support Group members, students, and faculty from two medical schools, along with members of Voice of the Experienced (VOTE)–FIP who have successfully navigated the reentry period–co-constructed a trauma-informed peer support curriculum with the implicit goal of undoing the effects of long-term institutionalization. The curriculum was developed through a series of focus groups identifying major topics of importance for life after incarceration. The initial topics were wide-ranging, given the numerous struggles FIP face upon reentry. Early discussions focused on prioritizing key topics and then grouping related topics together into individual units. Successive iterations were refined by breakout teams of medical and public health students partnered with FIP. These teams identified and prioritized topics related to release that were conducive to FIPS Group’s vision of a mutual support group. They would then regroup with academic consultants with experience in curriculum design.

The final list of topics, depicted in Table 1, range from navigating family dynamics to substance use, to processing the “culture shock” of returning to homes (for some) and new communities after decades of incarceration. The final curriculum consists of an orientation session, 12 units of the highest priority topics, and a graduation celebration. Each unit includes a background rationale, learning objectives, facilitation notes and discussion prompts. An example of the unit on Parenting and Family can be found in the Supplementary material. Detailed information of the intervention content is available from the corresponding author. Given the broad range of topics and duration of the curriculum, participants are invited to join in at any point in the curriculum and encouraged to attend units they missed previously in future sessions. Further, given that many topic discussions may overlap, participants who achieved a target dose of 75% attendance, or eight of the 12 units, are considered to have completed the curriculum.

**TABLE 1:** List of final curriculum topics used in 2020 and 2021 FIPS group.

Culture shock
Poverty and money issues
Jobs and conflict with authority
Goals, planning, time management, and responsibility
Parenting and family
Dating and relationships
Stigma and profiling
Substance use vs. abuse
Spirituality and meaning
Physical health
Mental health

## Implementation

The pilot curriculum was launched in 2020. Initially, the group continued to meet in-person, twice a month, for 2-h in the early evening. Participant recruitment was primarily through word-of-mouth from TT himself, other attendees, or local reentry programs. Additionally, FIT Clinic patients were provided brochures with FIPS Group information. Most sessions began with 20 min of participants greeting each other and introducing themselves, followed by a brief overview of the session’s topic, why it matters, why it is relevant to FIP specifically, and the facilitator’s personal experiences with the subject. Then, the facilitator calls on individual attendees to give their perspective on the topic, ensuring no one speaks over anyone else, redirecting conversations that get lost in tangent, and offering validation and encouragement when needed.

The COVID-19 pandemic created a challenge. Recognizing that peer support could not be interrupted, the FIPS Group quickly pivoted to a virtual format, offering sessions via Zoom. This initially posed some setbacks, particularly due to members’ inexperience with technology after years of incarceration. However, with the assistance of students and volunteers, FIPS Group was able to overcome these barriers, and ultimately saw increased attendance. The virtual format allowed for participation from FIP living in multiple states, including Indiana, Illinois, California, and Georgia, in addition to other parts of Louisiana. The FIPS Group now includes a network of more than 150 stakeholders, from participants, referral organizations, community partners, academic centers, clinicians, student volunteers, advocacy groups, and more.

Though the increase in participants has been welcome, and indicates the demand for spaces for FIP, it has compelled the organizing team to strategize means of ensuring integrity of content delivery and promoting inclusive peer participation. As such, the facilitator and participants identified optimal attendance of around 12–15 FIP to have the best means of letting all FIP participants contribute to discussion. To avoid any limitation on attendance, the facilitator makes special effort to ensure every FIP responds at least once during the session. Participation of family and loved ones is still encouraged as it was during in-person sessions.

## Program development outcomes

During the 2020 curriculum pilot, attendance data collection was disrupted through the pandemic and is thus incomplete. However, from the six recorded sessions, there was a total attendance of 34 FIP who attended at least one session. The average FIP attendance per session was 10.17 (SD = 3.87), or 29.9% of total FIP participants. Though attendance data collection was prioritized as a part of the formalization proceeding from the 2020 pilot, these data are underestimates due to multiple participants frequently join from the same device.

The 2021 iteration of the FIPS Group curriculum had a total attendance of 75 FIP who attended at least one session. Average FIP attendance per session was 15.42 (SD = 5.87), or 20.6% of total FIP participants. Regarding retention rate, 24% of those who participated in the 2020 curriculum returned for the 2021 program.

Regarding completion rate, as depicted in Table 2, around 9% of potential 2021 FIP attendants completed half or more of the units, and only 5.3% completed 10–12 units. Only 6.67% of attendees achieved target completion dose within the 2021 curriculum. Given the growing number of contacts within FIPS Group’s network and need to increase completion rates, we identified limitations with email communication of meeting details as a low-yield form of recruitment and retention, pivoting toward automated text reminders, in addition to the traditional word-of-mouth network. Following this change, retention rates increased from 24% in the 2020–2021 period, to 40.4% in the 2021–2022 period to date.

**TABLE 2:** Attendance in 2021 curriculum.

Number of units attended	Number of participants (% of total)
1–3	58 (77.33)
4–6	10 (13.33)
7–9	3 (4.00)
10–12	2 (5.33)

Given that FIPS group’s curriculum is designed to be modular, allowing a jump-in, jump-off approach over time, the cumulative completion rates were identified as a better metric of success. Over the total period from 2020 to 2022 to date, there have been a total of 125 FIP participants, with a cumulative completion rate of 10.4%. The cumulative retention rate over the same period has been 8.8%.

## Discussion

### Program feasibility

The feasibility of the FIPS Group model hinges on its highly collaborative and iterative processes that center formerly incarcerated voices at every step. The organic nature of its development is its greatest strength, and the shared ownership among FIP is the cornerstone of its sustainability. Unlike other peer support programs initiated by clinical, institutional, or professional parties, the FIPS Group curriculum was made by FIP, for FIP. Such an orientation is summarized in our motto “Us Helping Us.” In this way, FIPS Group may be most comparable to peer support groups for veterans, where recognizing their unique challenges are strongly associated with shared experiences in a total institution (45, 46). These factors were the key elements leading to the development of the formal curriculum.

Since the onset of the COVID-19 pandemic in 2020, FIPS Group has been conducted over Zoom. This adaptation of the group space has had unforeseeable consequences and benefits for the program. Since participation now depends on access to the internet and email, the transition to Zoom has enabled multistate participation from FIP who moved out of Louisiana after leaving Angola. Additionally, several FIP who were incarcerated in other states have participated in the group, enriching the curricular discussions with experiences from other penal systems. Finally, some attendees note that the transition to Zoom is more convenient for their work schedules and allows their families and spouses to listen in and learn alongside them when they log on from home. However, the limited technological proficiency of many participants has resulted in limited engagement for some long-time members. Developing a sustainable digital literacy support role for student volunteers has helped facilitate the growth of the program.

### Considerations for evaluating programs with formerly incarcerated people

The nature of FIPS Group as a space makes formalization and evaluation in the traditional sense challenging. Many group participants were understandably reticent about being used as “guinea pigs” in any kind of outcome evaluation of the effects of the curriculum. Nevertheless, ongoing inclusive dialogue regarding a forthcoming qualitative program evaluation has eased these reservations. The role of the facilitator was key in mediating between the professional expertise involved in both curricular development and evaluation, as well as prioritizing the integrity of FIPS Group as a private space. Through these dynamics, generalizing the FIPS Group model requires the direction of organic leaders identified among local formerly incarcerated community members, to generate buy-in and ensure that shared decision-making and power-sharing are maintained. These values are most consistent with a community-based participatory action research (CBPAR) model of program development and evaluation (46).

An important consideration for working with those who have been incarcerated are norms of inclusion and respect, especially when academic partners broach discussions of monetary incentives for participation. Given the nature of being exploited by the carceral state, FIP are highly sensitive to any perception of being used. Many in the group had encounters with academicians and non-profits who would pay them for discrete projects and then leave them in the dark at subsequent steps. Therefore, a financial incentive alone is insufficient: having an inclusive and transparent process when working with FIP is critical and just as important as any product of the collaboration. Researchers who wish to work with those impacted by incarceration must prioritize partnership and community with FIP. This praxis is essential to overcoming the institutional abuse and neglect that have been the historic norm for this population.

### Outcomes of interest

In addition to traditional outcome measures, which may be difficult to study within the flexible and voluntary framework of the FIPS group, the authors have found process measures to be the most feasible and useful for evaluating the current program at the outset. Including short feedback surveys at the end of the emails containing the Zoom link for each session may offer participants the opportunity to anonymously report reasons for their absence as well as any general suggestions for the planning team. The results of these surveys may be reviewed after each session. Other process outcome measures that may be feasibly studied include percentage of participants who are formerly incarcerated (versus allies, family members or other loved ones), the average amount of time since attendees’ most recent release from incarceration, number of sessions provided, percentage of attendees who complete a feedback survey, and compliance with the original protocol (e.g., asking or discussing all intended sample questions) on a module-by-module basis.

One important cognitive outcome measure identified by planning for the forthcoming qualitative evaluation assesses participants’ reasons for attending and how it changed over time. More specifically, the authors are interested in the proportion of participants at the end of the curriculum whose main reason for attending sessions were descriptions of therapeutic benefit. For example, participants may have initially attended just to please another attendee or our facilitator who invited them, or out of curiosity, but over time, became motivated by therapeutic benefit (e.g., emotional support, to learn lessons from other attendees, or to connect with a FIP community) – a positive indicator of the group’s impact.

Moving forward, future evaluations may take a quantitative approach by posing a multiple-response survey question, “What is your main reason for joining FIPS?” including answer choices such as “To get emotional support,” “Curiosity,” etc. Researchers could then compare the number of selections between the first-time participants took the survey and by the time of the last session. The qualitative study also asks about times participants have reflected on discussions outside of group, ways that their relationships outside of group may have been impacted by group discussions, and how attending group has affected their self-understanding.

### Formerly incarcerated peer support group in the context of reentry experiences

The FIPS Group curriculum is unique in acknowledging that many of the ongoing challenges FIP face are rooted in the traumatic and institutionalizing effects of incarceration, as embodied by Mr. J. For example, although finding employment is often the goal of reentry programs, and many FIP are able to get jobs, (47) institutionalized coping strategies related to respect and boundaries are often reasons for difficulty keeping a job. By discussing, normalizing, and understanding both shared and alternative narratives via others’ stories after release, the FIPS Group curriculum addresses this root cause. Participants can identify difficulties and discover adaptive behaviors and coping strategies through group learning.

Formerly Incarcerated Peer Support Group actively discusses the concrete policies and cultural challenges posed by the carceral state that entrench social exclusion after release, with the intent of overcoming cultural gaslighting and achieving empowerment. As constructed by Ruíz, cultural gaslighting can be defined as the social and historical infrastructural support mechanisms that disproportionately produce abusive mental ambients in settler colonial cultures to further the ends of cultural genocide and dispossession (48). The application to FIP is apt. Dominant narratives of “second chances” in cultural overtures conflict with FIP’s experience of distrust, disdain, and expectations of from those who have not been incarcerated. These negative perceptions become more likely through thousands of policies collectively termed collateral consequences of incarceration, including ineligibility for many professional licenses, being banned from public housing, electoral disenfranchisement, rejection from education opportunities, among many others (49). Consequently, many FIP anticipate and experience stigma in their social lives, at work, and in reentry programs and subsequently respond with internalized self-stigma, feeling anguish at being unwanted and insufficient, coping through withdrawal and isolation (50–52). The tenor of the formerly incarcerated community, unspoken in most public settings, is a disdain toward government and rejection of the validity of their ongoing punishment, rather than accepting accountability and moving forward.

If, in fact, FIP feel alienated in a society that does not want formerly incarcerated people, then they are being objectively realistic, and without a community touchstone for this systemic oppression, an individual has few outlets for their negative emotions. However, at the opposite end of the stigma continuum, as discussed by Corrigan and Rao, is an “enhanced sense of empowerment” that comes from peer support groups like FIPS group (50). The antidote to social exclusion is a community that can acknowledge and dismantle oppressive systems. Thus, FIPS Group offers members a space to validate their experiences while gaining empowerment in a non-stigmatized zone, which, in turn, helps them navigate the dehumanizing policies and narratives that stigmatize FIP.

## Limitations

The present paper is not a formal evaluation of the curriculum, but rather offers insight into the decisions that guided the group’s programmatic development. As such, the authors sought to provide an example of the processes for those seeking to implement similar peer-led interventions. A formal CBPAR-oriented qualitative evaluation is currently underway to assess perceptions of the curriculum and understand how peer support has affected the reintegration process for FIP.

An additional limitation is that FIPS Group represents a single community of FIP. Though it has grown to include a multistate participant base, most participants are part of a network formed at a single prison. Most of the regular participants were also released years ago, and therefore may not experience as much instability and mental distress as someone released recently. Implementing additional outreach efforts, and wider referral networks remain a focus of FIPS Group to ensure that those who may benefit most from a supportive community can participate.

An area for future growth is defining a similar space for formerly incarcerated women. The overwhelming majority of both the planning team, including the facilitator, and the group itself, are men. As a result, the program is not sensitive to the unique experiences of the few formerly incarcerated women in the group. For example, the curriculum was ill-equipped to engage in a discussion about single motherhood during reentry, reproductive healthcare during and after incarceration, the struggles of dealing with sexual trauma, and many more experiences that are more common among formerly incarcerated women than men. To fill this gap, many of those who collaborated on FIPS Group are developing a pilot peer support group to create an analogous, but population-specific space for formerly incarcerated women to gather in solidarity.

Finally, a broader understanding of qualitative reentry experiences would be bolstered by contextualizing FIP within the perspectives of the surrounding society. Research on the attitudes and actions of employers, neighbors, families, academia, landlords, elected officials, and others should be both objective and subjective. By analyzing both perspectives in tandem, the most impactful interventions may be designed and implemented.

## Conclusion

The FIPS Group is an organic FIP-led response to filling a gap in much-needed community-specific mental health services for FIP. Its development has been highly collaborative and flexible, enabling sustainable growth. Traditional reentry programs have presumed the needs of FIP and have only recently recognized the value of building peer networks, while FIPS Group’s orientation is toward community building, undoing institutionalization, and promoting inclusion. The focus on FIP-identified root causes, rather than tangential institutional concerns may be expected to create a cascade of positive interconnectedness and mutual support that can be replicated in an “each one teach one” manner. This is a versatile, high-impact program that may be generalized in a number of settings to benefit FIP. Similar approaches to developing peer-led resources may enhance the mental health of millions of Americans who, like Mr. J, were affected by the criminal-legal system and then returned, alone, to an unfamiliar place they once called home.

## Author’s note

Ashley Wennerstrom and Bruce Reilly are Robert Wood Johnson Foundation Interdisciplinary Research Leaders Fellows.

## Data availability statement

The original contributions presented in this study are included in the article/Supplementary material, further inquiries can be directed to the corresponding author.

## Author contributions

TT, AW, AN, and BR contributed to conception and design of the program. JW, LN, AV, and WB managed logistics of focus groups and curricular development. TT and JW delivered the program content. AS and CM revised program manual and supported subsequent program logistics. WB wrote the first draft of the manuscript. WB, TT, JW, AW, BR, and AN wrote sections of the manuscript. All authors contributed to manuscript revision, read, and approved the submitted version.
