# Supplementary figures and images for: Us helping us: The evolution of a peer support group for formerly incarcerated people
Source: Front Psychiatry. 2022 Aug 2;13:920640. doi: 10.3389/fpsyt.2022.920640 (PMC9379313; doi:10.3389/fpsyt.2022.920640)

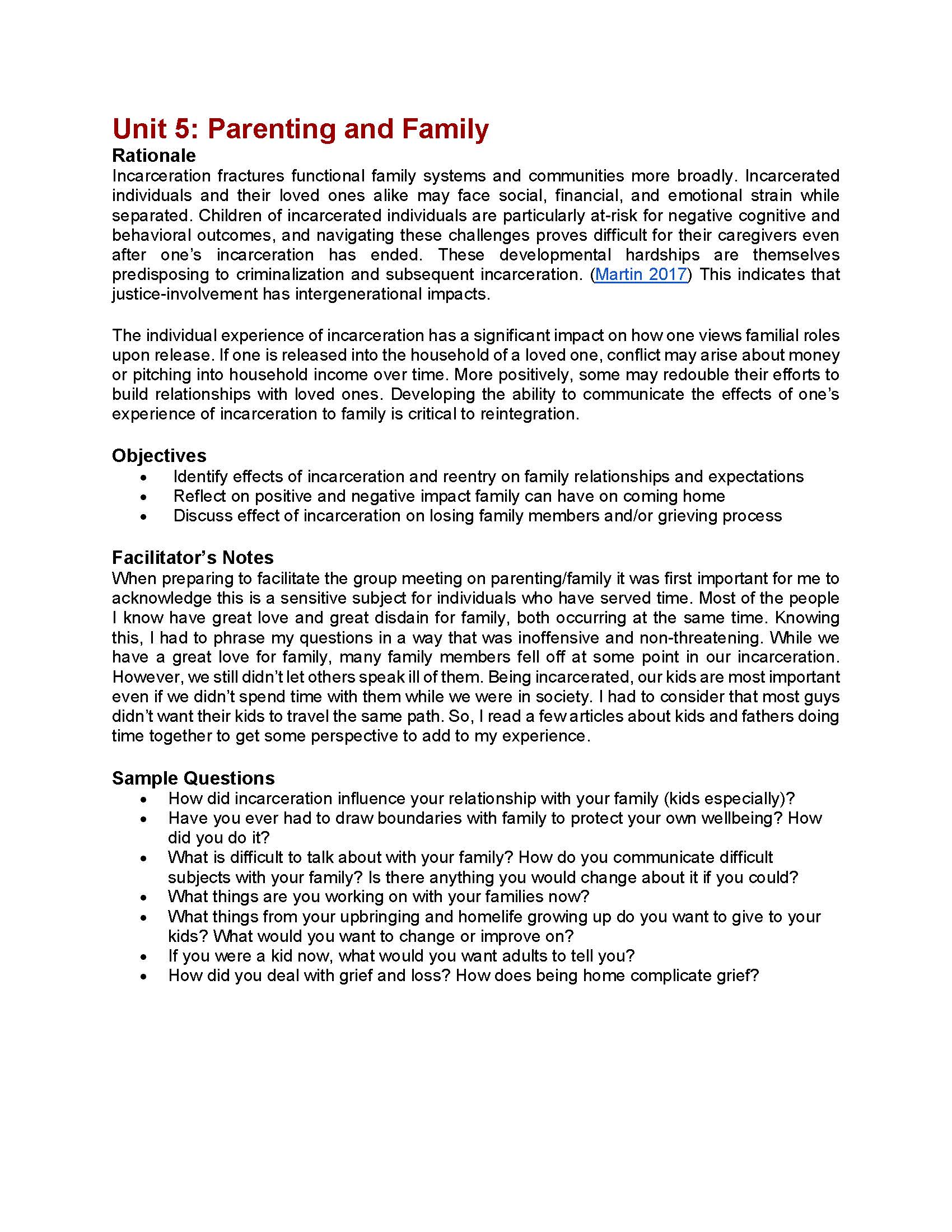

Supplement: Supplementary file 1 [file Image_1.JPEG]
